# Efficacy and safety of IL-23 inhibitors in the treatment of psoriatic arthritis: a meta-analysis based on randomized controlled trials

**DOI:** 10.1007/s12026-023-09366-4

**Published:** 2023-02-22

**Authors:** Xiaojing Huang, Haojie Shentu, Yujing He, Haijia Lai, Chen Xu, Meiling Chen, Haowei Zhu

**Affiliations:** 1 Emergency Medical Center, Ningbo Yinzhou No. 2 Hospital, 998 North Qianhe Road, Yinzhou District, Ningbo, Zhejiang, 315100 China; 2 School of Medical Imaging, Hangzhou Medical College, Zhejiang, Hangzhou China; 3 The Second Clinical Medical College, Zhejiang Chinese Medical University, Zhejiang, Hangzhou China; 4 School of Nursing, Hangzhou Medical College, Zhejiang, Hangzhou China; 5 The Public Health College, Zhejiang Chinese Medical University, Zhejiang, Hangzhou China

**Keywords:** IL-23 inhibitors, Psoriatic arthritis, Biologics, Randomized controlled trials, Meta-analysis

## Abstract

In recent years, the use of interleukin (IL) 23 inhibitors in the treatment of psoriatic arthritis (PsA) has been the subject of much research. By specifically binding to the p19 subunit of IL-23, IL-23 inhibitors block downstream signaling pathways and inhibit inflammatory responses. The objective of this study was to assess the clinical efficacy and safety of IL-23 inhibitors in the treatment of PsA. PubMed, Web of Science, Cochrane Library, and EMBASE databases were searched from the time of conception to June 2022 for randomized controlled trials (RCTs) investigating the use of IL-23 in PsA therapy. The main outcome of interest was the American College of Rheumatology 20 (ACR20) response rate at week 24. We included six RCTs (3 studies on guselkumab, 2 on risankizumab, and 1 on tildrakizumab) with a total of 2971 PsA patients in our meta-analysis. We found that the IL-23 inhibitor group showed a significantly higher ACR20 response rate compared to the placebo group (relative risk = 1.74, 95% confidence interval: 1.57–1.92; *P* < 0.001; *I*^2^ = 40%). There was no statistical difference in the risk of adverse events (*P* = 0.07) and serious adverse events (*P* = 0.20) between the IL-23 inhibitor and placebo groups. Notably, the rate of elevated transaminases in the IL-23 inhibitor group was higher than the placebo group (relative risk = 1.69; 95%CI 1.29–2.23; *P* < 0.001; *I*^2^ = 24%). In the treatment of PsA, IL-23 inhibitors significantly outperform placebo intervention while maintaining a favorable safety profile.

**Supplementary Information:**

The online version contains supplementary material available at 10.1007/s12026-023-09366-4.

## Introduction

Psoriatic arthritis (PsA) is an immune-mediated inflammatory joint disease that affects 20–30% of people with psoriasis [1]. There are several clinical and radiographic manifestations of PsA, including arthritis, enthesitis, axial disease, and involvement of the skin and nails [2, 3]. Studies have shown that PsA imposes a significant financial burden on patients, with an average annual healthcare cost of nearly $30,000 per patient [4, 5]. At the same time, patients with PsA also experience significant psychosocial burdens [6].

Treatment of PsA varies according to the severity of the condition. The mainstay treatment strategy for patients with a mild oligoarticular presentation were nonsteroidal anti-inflammatory drugs (NSAIDs) and conventional synthetic disease-modifying antirheumatic drugs (csDMARDs). In contrast, a biological disease-modifying antirheumatic drug (bDMARD) should be used in patients with severe peripheral arthritis who have not responded to at least one csDMARD [7, 8]. Currently available bDMARDs include tumor necrosis factor (TNF) inhibitors, interleukin (IL)12/23 inhibitors, IL-17 inhibitors, and IL-23 inhibitors. Although IL-23 inhibitors are comparatively newer and further research is still in progress, some studies have confirmed their safety and efficacy [9–11].

IL-23 inhibitors approved for the treatment of PsA include guselkumab, tildrakizumab, and risankizumab. The use of IL-23 inhibitors may provide additional treatment options for patients who have not been adequately managed with standard therapies. Numerous randomized controlled trials (RCTs) have been conducted in recent years to substantiate the clinical efficacy and safety of IL-23 inhibitors. In the present study, IL-23 inhibitors were further evaluated for efficacy and safety via a meta-analysis based on published RCTs.

## Methods

### Search strategy

This study was carried out according to Preferred Reporting Items for Systematic review and Meta-Analysis (PRISMA) guidelines [12]. From their earliest records to June 2022, PubMed, Web of Science, Cochrane Library, and EMBASE databases were searched for RCTs comparing IL-23 inhibitors with placebos or other active treatments for PsA. The IL-23 inhibitors of interest included guselkumab, tildrakizumab and risankizumab. The search algorithms included the following terms: (“Interleukin 23” OR “IL-23” OR “Risankizumab” OR “Tildrakizumab” OR “Guselkumab”) AND (“Psoriasis, arthritis” OR “Arthritic Psoriasis” OR “Psoriatic Arthritis” OR “Psoriasis Arthropathic” OR “Psoriatic Arthropathy” OR “Arthropathies, Psoriatic” OR “Arthropathy, Psoriatic” OR “Psoriatic Arthropathies”).

### Study inclusion and exclusion criteria

The “PICOS” principle was used throughout the study to screen and exclude articles. Selected articles were further subjected to full-text analysis. Studies that met the following criteria were considered eligible: (a) the study population was PsA patients aged 18 years or older; (b) the intervention was IL-23 inhibitors and comparison were placebo and/or other treatments; (c) the outcomes included American College of Rheumatology 20 (ACR20, defined as at least a 20% improvement in the American College of Rheumatology response criteria [13]), ACR50, ACR70, and drug-related adverse events; (d) the study type was phase II/III RCTs.

Studies not meeting any of the following criteria were excluded: (a) the language of the article is not English; (b) data from the study were not extractable or missing; (c) use of biologics other than IL-23 inhibitors during treatment; (d) if studies were derived from the same participants, the most recent studies will be evaluated.

### Data extraction

Data from each study were extracted independently by two reviewers using standardized formats, and any discrepancies were resolved through discussion. The following data was documented: author, year of publication, number of patients, sex, average age, treatment regimen, reported outcomes, and AEs.

The primary outcome was the number of patients who achieved ACR20 at week 24. The secondary outcomes included ACR50, ACR70, Psoriasis Area Severity Index (PASI) 75, and/or PASI 90.

### Quality evaluation and outcome measures

Cochrane Collaboration’s tool [14] was used to assess the inherent risk of bias of included RCTs. The criteria used to assess bias included seven aspects: (a) random sequence generation (selection bias), (b) allocation concealment (selection bias), (c) blinding of participants and personnel (performance bias), (d) blinding of outcome assessment (detection bias), (e) incomplete outcome data (attrition bias), (f) selective reporting (reporting bias), (g) other biases. Each of the aspects was scored as “low risk,” “high risk,” or “unclear risk,” and low overall risk indicates that the study is less prone to bias.

### Statistical analysis

Differences in outcomes between groups are represented using relative risk (RR) and corresponding 95% confidence interval (CI). Heterogeneity between studies was assessed using the *I*^2^ statistic, which represents the reliability of the result analysis: A value of 0% indicates no observed heterogeneity, and larger values show increasing heterogeneity [15]. When the heterogeneity of results was high (*I*^2^ > 75%), pre-defined subgroup analyses were established to identify sources of heterogeneity. If more than 10 studies were included, publication bias and sensitivity analyses were performed. Because three different IL-23 inhibitors were included in this study, a random-effects model (DerSimionan and Laird method) [16] was used to analyze the results of the included studies. A two-sided *P*-value < 0.05 for all the above statistical results was considered statistically significant. RevMan version 5.4 software was used for all statistical analyses.

## Results

### Literature search results

**Fig. 1:**
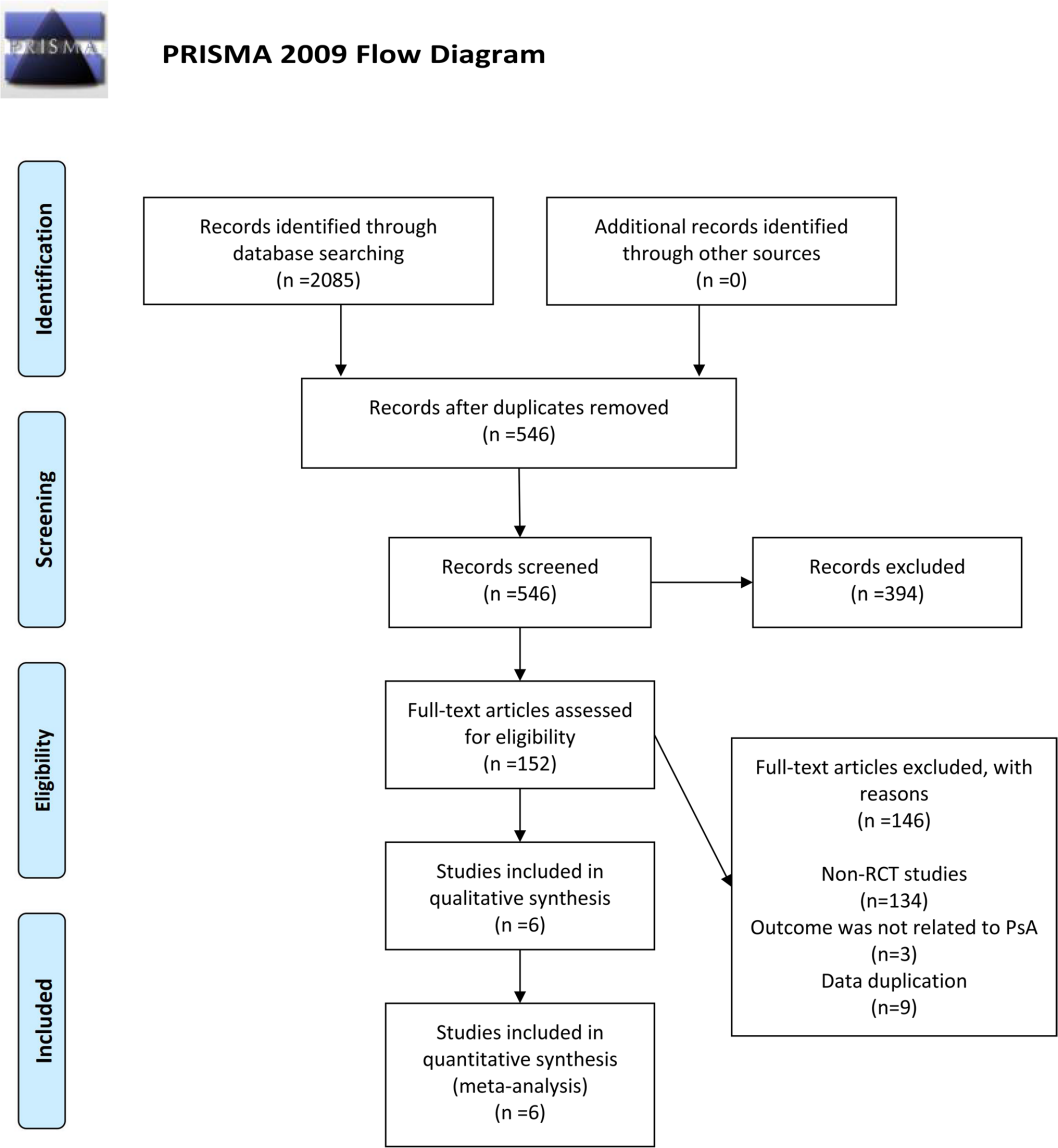
Flow diagram describing inclusion and exclusion criteria

The literature search identified 2085 citations based on our search strategy. After excluding duplicates, the title and abstract of the remaining 546 citations were reviewed, and 394 were excluded for deviation from the inclusion criteria. Of the remaining 152 articles, 146 were excluded after reading the full text, of which 134 were excluded due to being non-RCTs, 9 were eliminated because of duplication, and 3 were excluded because they were unrelated to PsA. In total, 6 articles were included in this meta-analysis. The specific screening and exclusion processes are shown in Fig. 1.

### Study characteristics and quality

The six included studies were all RCTs investigating the efficacy of IL-23 inhibitors in the treatment of PsA, of which three used guselkumab [17–19] (including 1173 patients), two used risankizumab [20, 21] (including 1407 patients), and one used tildrakizumab [22] (including 391 patients). All included studies were placebo-controlled, and all studies reported on safety outcomes, ACR20 response rates at week 24, as well as ACR50, ACR70, and PASI90 response rates.

The baseline characteristics of the included RCTs are detailed in Table 1. The mean duration of PsA history in patients across studies ranged from 5.1 to 8.2 years. The mean number of swollen joint counts ranged from 9.4 to 13.6, and the mean number of tender joint counts ranged from 16.6 to 22.8. In five trials [17–21], 61.5–76% of patients had symptoms of enthesitis, and 17.9–61% of patients reported dactylitis. In two trials [18, 20], patients had never been exposed to biologics previously. All studies included patients who had previously used disease-modifying antirheumatic drugs (DMARDs) and methotrexate. The proportion of patients who have used DMARDs and methotrexate is shown in Supplementary Table 1.

**Table 1:** Baseline characteristics of the included RCTs in the meta-analysis

Author, year	Therapeutic schedule	Average age (years)	Male	Duration of PsA, years	PASI score (0–72)	Swollen joint count (0–66)	Tender joint count (0–68)
Deodhar, Atul 2018	Guselkumab 100 mg at week 0, week 4, and every 8 weeks	47	52%	7.0 ± 7.2	12.0 ± 10.5	11.9 ± 7.6	20.7 ± 12.2
	Placebo	44	24%	6.9 ± 7.2	9.9 ± 8.0	10.6 ± 7.5	20.1 ± 12.5
Mease, Philip J. 2020	Guselkumab 100 mg every 4 weeks	46	58%	5.5 ± 5.9	10.8 ± 11.7	12.9 ± 7.8	22.4 ± 13.5
	Guselkumab 100 mg at weeks 0, 4, then every 8 weeks	45	52%	5.1 ± 5.5	9.7 ± 11.7	11.7 ± 6.8	19.8 ± 11.9
	Placebo	46	48%	5.8 ± 5.6	9.3 ± 9.8	12.3 ± 6.9	21.6 ± 13.1
Coates, L. C. 2022	Guselkumab 100 mg week 0, week 4, then every 8 weeks	49	46%	8.3 ± 7.8	11.7 ± 11.9	10.0 ± 7.0	21.0 ± 13.0
	Placebo	49	54%	8.7 ± 7.2	9.2 ± 9.4	9.0 ± 6.0	18.0 ± 11.0
Kristensen, L. E. 2022	Risankizumab 150mg at weeks 0, 4 and 16	52	52%	7.1 ± 7.0	10.9 ± 10.1	12.1 ± 7.8	20.8 ± 14.1
	Placebo	52	49%	7.1 ± 7.7	10.0 ± 10.4	12.2 ± 8.0	20.5 ± 12.8
Ostor, A. 2022	Risankizumab 150mg at weeks 0, 4 and 16	53	45%	8.2 ± 8.2	7.7 ± 6.7	13.0 ± 8.7	22.8 ± 14.9
	Placebo	52	45%	8.2 ± 8.3	8.4 ± 9.9	13.6 ± 9.0	22.3 ± 13.8
Philip J Mease 2021	Tildrakizumab 200 mg every 4 weeks	50	41%	7.5 ± 8.5	7.6 ± 9.8	10.4 ± 7.4	16.6 ± 11.9
	Tildrakizumab 200 mg every 12 weeks	49	53%	6.2 ± 7.2	6.2 ± 7.4	10.0 ± 8.0	19.5 ± 13.9
	Tildrakizumab 100 mg every 12 weeks	49	39%	7.0 ± 6.6	8.8 ± 9.5	11.0 ± 8.2	21.3 ± 14.8
	Tildrakizumab 20 mg every 12 weeks	47	47%	6.6 ± 6.7	5.5 ± 2.1	9.4 ± 6.4	19.0 ± 13.0
	Placebo	48	44%	6.3 ± 6.1	5.0 ± 6.5	11.8 ± 9.8	19.7 ± 14.7

*RCT*, randomized controlled trial; *PsA*, psoriatic arthritis; *PASI*, Psoriasis Area Severity Index

The six studies were appraised for quality using Cochrane Collaboration’s tool, and all trials were judged to be at a low inherent risk of bias. Figs. S1 and S2 show the specific quality assessment results.

### Efficacy analysis

**Fig. 2:**
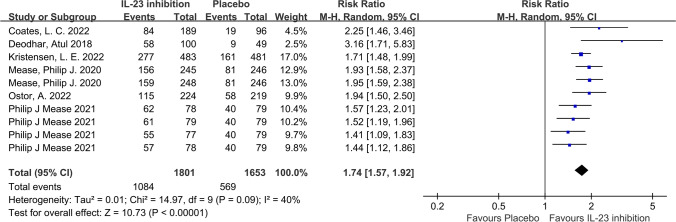
Forest plot of interleukin-23 inhibitors versus placebo in ACR20

**Fig. 3:**
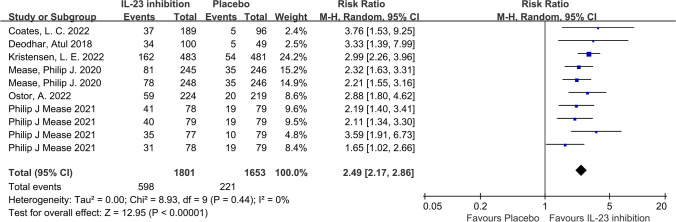
Forest plot of interleukin-23 inhibitors versus placebo in ACR50

**Fig. 4:**
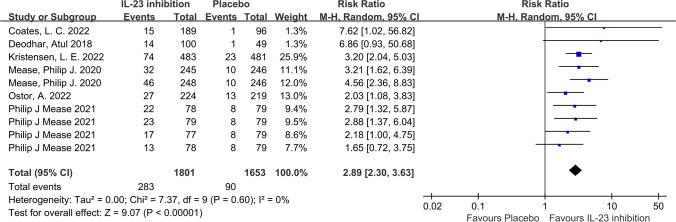
Forest plot of interleukin-23 inhibitors versus placebo in ACR70

**Fig. 5:**
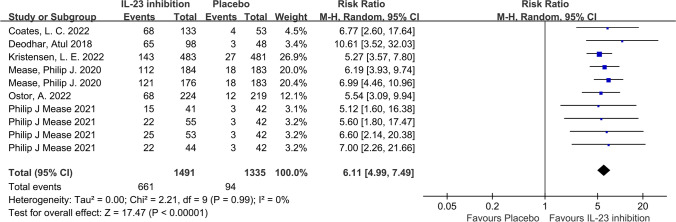
Forest plot of interleukin-23 inhibitors versus placebo in PASI90

**Fig. 6:**
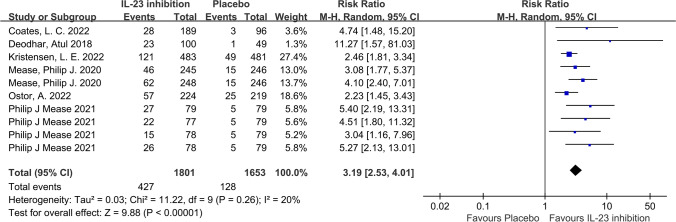
Forest plot of interleukin-23 inhibitors versus placebo in minimal disease activity

In terms of our primary outcome, ACR20, the IL-23 inhibitor group showed significantly higher response rates compared to the placebo group, and the pooled RR across the 6 trials was 1.74 (95%CI: 1.57–1.92; *P* < 0.001; *I*^2^ = 40%; Fig. 2). Regarding secondary outcomes, the IL-23 inhibitor group also showed a significantly higher ACR50 response rate (RR = 2.49; 95%CI: 2.17–2.86; *P* < 0.001; *I*^2^ = 0%; Fig. 3) and ACR70 response rate (RR = 2.89; 95%CI: 2.30–3.63; *P* < 0.001; *I*^2^ = 0%; Fig. 4) than the placebo group. Moreover, in terms of skin relief, the rate of patients achieving PASI90 in the IL-23 inhibitor group was significantly higher than in the placebo group (RR = 6.11; 95%CI: 4.99–7.49; *I*^2^ = 0%; *P* < 0.001; Fig. 5). Furthermore, the minimal disease activity (MDA, which represent the disease activity states that both patients and physicians consider being an effective target for treatment [23]) response rate of the IL-23 inhibitor group was also significantly higher than the placebo group (RR = 3.19; 95%CI: 2.53–4.01; *P* < 0.001; *I*^2^ = 20%; Fig. 6).

**Fig. 7:**
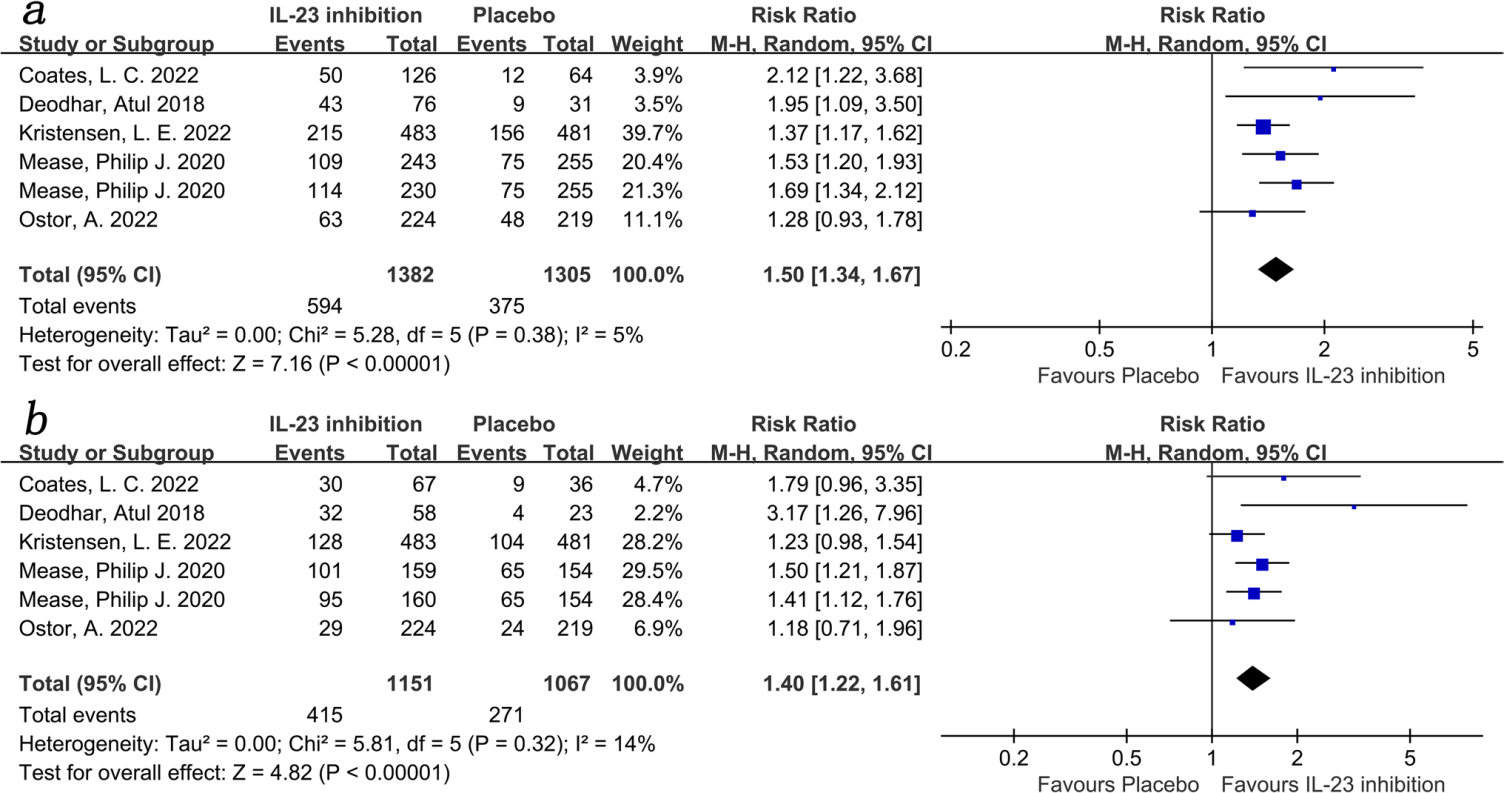
Forest plot of interleukin-23 inhibitors versus placebo in enthesitis and dactylitis (*a* enthesitis; *b* dactylitis)

Enthesitis and dactylitis are clinical manifestations of PsA and reflect disease severity [2]. Five trials [17–21] reported the number of patients with dactylitis and enthesitis. The results showed that more people in the IL-23 inhibitor group recovered from enthesitis (RR = 1.50; 95%CI: 1.34–1.67; *P* < 0.001; *I*^2^ = 5%; Fig. 7a) and dactylitis (RR = 1.40; 95%CI: 1.22–1.61; *P* < 0.001; *I*^2^ = 14%; Fig. 7b) compared to the placebo group.

### Safety analysis

The safety outcomes of the six studies are represented in Table 2. The common adverse reactions were nasopharyngitis, upper respiratory tract infection, and elevated transaminases. Nasopharyngitis, upper respiratory tract infection, adverse events (AEs), and serious adverse events (SAEs) were reported in all studies. However, one study's data on elevated transaminases and infections were absent [22]. In terms of AEs (RR = 1.07; 95%CI: 0.99–1.15; *I*^2^ = 0%; *P* = 0.07) and SAEs (RR = 0.78; 95%CI: 0.52–1.15; *I*^2^ = 0%; *P* = 0.20), there was no statistical difference in the incidence between the IL-23 inhibitors and placebo groups. There was also no difference between both groups regarding infection incidence, upper respiratory tract infection, and nasopharyngitis. Notably, patients in the IL-23 inhibitor group had a higher rate of elevated transaminases compared to the placebo group (RR = 1.69; 95%CI 1.29–2.23; *P* < 0.001; *I*^2^ = 24%).

**Table 2:** Adverse events for the IL-23 inhibition and placebo

IL-23 inhibition vs. placebo	No. of studies	Participants	Risk ratio	95%CI	*p*	Heterogeneity (*I*^2^) (%)
Patients with more than adverse event	6	2971	1.07	0.99–1.15	0.20	0
Patients with more than serious adverse event	6	2971	0.78	0.52–1.15	0.07	0
Elevated transaminases	5	2580	1.69	1.29–2.23	< 0.001	24
Infections	5	2580	0.97	0.74–1.26	0.81	62
Upper respiratory tract infection	6	2971	1.09	0.76–1.56	0.66	0
Nasopharyngitis	6	2971	1.03	0.75–1.42	0.84	0

## Discussion

There has been an increase in the use of IL-23 inhibitors in the treatment of PsA over the past few years. Herein, a meta-analysis of 6 RCTs was conducted to examine the efficacy and safety of IL-23 inhibitors (guselkumab, risankizumab, tildrakizumab) in the treatment of PsA. To date, there has been no systematic review or meta-analysis investigating IL-23 inhibitors for the treatment of PsA to the best of our knowledge.

IL-23, a cytokine involved in inflammation and autoimmunity, is a heterodimer composed of two subunits, p19 and p40, which can bind to the IL-23 receptor on T-helper 17 (Th17) cells and drive the expansion of Th17 cells to produce more pro-inflammatory factors to trigger joint and synovial inflammation. It has proven essential in the induction of arthritis, osteoclastic formation, and maintenance of bone mass [24]. IL-23 inhibitors selectively bind to the p19 subunit of IL-23 with high specificity and affinity, therefore, the IL-23 signaling pathway was blocked, the proliferation of Th17 cells was inhibited and the symptoms of PsA improved [7, 18, 25].

This meta-analysis confirmed that IL-23 inhibitors, guselkumab, risankizumab, and tildrakizumab, can effectively treat PsA. Patients treated with IL-23 inhibitors exhibited significantly improved ACR20, ACR50, ACR70, and PASI90 scores compared to those receiving the placebo intervention. In addition, IL-23 inhibitors were also effective in relieving enthesitis and dactylitis and improving quality of life (MDA). The in-depth study of the pathogenesis and treatments of PsA has allowed us to understand that dysregulation of the IL-23/IL-17 axis can greatly facilitate the induction of chronic inflammation and autoimmunity, and contribute to various autoimmune diseases such as PsA, psoriasis, ankylosing spondylitis, rheumatoid arthritis, inflammatory bowel disease, etc. [26–29]. Therefore, targeting the IL-17 and IL-23 signaling pathways can alleviate the symptoms of the disease. Existing biologics, such as TNF inhibitors (e.g., entanercept, influximab, adalimumab) were the first-generation drugs for PsA treatment, while IL-12/23 inhibitors (e.g., ustekinumab) and IL-17 inhibitors (e.g., secukinumab, ixekizumab) were the subject of research in the past few years. A head-to-head comparison of ixekizumab (an IL-17 inhibitor) and adalimumab (a TNF-alpha inhibitor) has shown that ixekizumab was associated with greater improvement in combined PsA and skin endpoints at 24 weeks [30]. In another head-to-head trial involving secukinumab (an IL-17 inhibitor) and adalimumab, secukinumab demonstrated higher clinical responses in terms of musculoskeletal endpoints, skin endpoints, and composite indices at week 52 [31]. As for the use of IL-12/IL-23 inhibitors in the treatment of PsA, a prospective randomized-controlled open-label study concluded that they could outperform TNF inhibitor treatment in terms of enthesitis remission rate [32]. Unlike IL-12/23 inhibitors, IL-23 inhibitors only specifically bind to the p19 subunit, preserving the activity of IL-12, which may improve its efficacy in PsA and psoriasis therapy [33–35]. The treatment guidelines of PsA have been updated in recent years as therapeutic options have increased. The Group for Research and Assessment of Psoriasis and Psoriatic Arthritis (GRAPPA) guidelines which have been recently updated, strongly recommend IL-23 inhibitors for PsA patients, while the recommendation for IL-23 use in patients with peripheral arthritis is weaker [36]. Interestingly, the most recent guidelines from the European League Against Rheumatism (EULAR) on PsA did not include a specific order for targeted bDMARDs use [37]. A study by Rahman et al. [38] indicated that guselkumab was more effective than TNF inhibitors in treating psoriatic skin, while showing comparable effectiveness in managing bronchitis, bunion, and PsA-related arthritis. Unfortunately, head-to-head clinical trials of IL-23 inhibitors versus other biologics are scarce, thus more clinical evidence is needed to support the efficacy of IL-23 inhibitors.

The results of our meta-analysis indicate that using interleukin-23 inhibitors did not significantly increase the risk of AEs. Consistently, the incidence of AE and SAEs observed in clinical trials between the IL-23 inhibitor and the placebo group were comparable. Compared to the placebo group, the number of patients with elevated transaminases in the IL-23 inhibitor group was significantly higher, but most reactions were grade one or grade two. A study by Proton et al. [38] obtained similar results and hypothesized that these elevations were associated with low and transient toxicity since it was more common in patients using methotrexate at baseline. In line with our findings, Loft et al. [10] found that the most common AEs during IL-23 inhibitor intervention were infection and nasopharyngitis, with significantly lower risks of infection and injection site reactions compared to IL-17 inhibitors use. In addition, multiple studies have reported an increased risk of infection following the use of IL-17 inhibitors because IL-17 and IL-22 play an important role in enhancing innate barrier defense on the mucosal surface, with disruption of IL-17 production or signaling increasing the risk for fungal and bacterial infections [28, 39]. Collectively, our analysis shows that IL-23 inhibitors have a robust safety profile.

Nevertheless, our meta-analysis has some limitations. Firstly, since the observation period was limited to 24 weeks, we could not assess the long-term safety of IL-23 inhibitors in PsA patients. Therefore, the incidence of adverse events from long-term IL-23 inhibitor use remains to be investigated. Secondly, due to the difference in study design, we did not compare the efficacy and safety of the three drugs (guselkumab, risankizumab, and tildrakizumab). Finally, due to limited data, subgroup analyses were not performed for the patients undertreated with TNF-alpha inhibitors, leaving uncertainty about the efficacy of IL-23 inhibitors in this subpopulation. Nonetheless, by including 6 high-quality phase 2/phase 3 RCTs and following a strict statistical analysis approach, this meta-analysis can provide a reference point for clinicians in the treatment of PsA.

## Conclusion

Overall, our results demonstrated that IL-23 inhibitors effectively improved joint disease activity in PsA patients while also having a good safety profile. IL-23 Inhibitors have the potential to be a promising therapeutic option, especially for PsA patients who have an insufficient response to csDMARDs. There are currently no head-to-head studies comparing IL-23 inhibitors with other biologics. Therefore, RCTs with larger sample sizes and longer durations are warranted to further validate the effectiveness of IL-23 inhibitors in PsA treatment.

## Supplementary information

Supplementary Table 1. Baseline characteristics 501 of the included RCTs in the meta-analysis

Fig. S1 Quality assessment of each study included in this meta-analysis

High resolution image (TIF 440 kb)

Fig. S2 Quality assessment of studies included in this meta-analysis (Risk of bias summary)

High resolution image (TIF 1105 kb)

## Data Availability

The datasets supporting this article’s conclusions are included within the article and its additional files.

## Abbreviations

PsA: psoriatic arthritis
RCTs: randomized controlled trials
IL: interleukin
ACR: American College of Rheumatology
AEs: adverse events
SAEs: serious adverse events
MAD: minimal disease activity
NSAIDs: nonsteroidal anti-inflammatory drugs
csDMARDs: conventional synthetic disease-modifying antirheumatic drugs
bDMARDs: biological disease-modifying antirheumatic drugs
TNF: tumor necrosis
PRISMA: Preferred Reporting Items for Systematic Review and Meta-Analysis
PASI: Psoriasis Area Severity Index
RR: relative risk
CI: confidence interval
GRAPPA: Group for Research and Assessment of Psoriasis and Psoriatic Arthritis
EULAR: European League Against Rheumatism
