# Supplementary material for: Efficacy and safety of IL-23 inhibitors in the treatment of psoriatic arthritis: a meta-analysis based on randomized controlled trials
Source: Immunol Res. 2023 Feb 22;71(4):505–15. doi: 10.1007/s12026-023-09366-4 (PMC10425519; doi:10.1007/s12026-023-09366-4)
Supplement: Supplementary file 1 — Baseline characteristics 501 of the included RCTs in the meta-analysis [file 12026_2023_9366_MOESM1_ESM.docx]

**Supplementary Table 1. Baseline characteristics of the included RCTs in the meta-analysis.**

| Author, year | Patients with enthesitis | | Patients with dactylitis | | Disease-modifying anti-rheumatic drugs Used | | Methotrexate Used | |
| --- | --- | --- | --- | --- | --- | --- | --- | --- |
|  | IL-23 inhibition | Placebo | IL-23 inhibition | Placebo | IL-23 inhibition | Placebo | IL-23 inhibition | Placebo |
| Deodhar, Atul 2018 | 76% | 63% | 58% | 47% | 90% | 84% | 47% | 39% |
| Mease, Philip J. 2020 | 69%; 64% | 72% | 49%; 45% | 40% | 69%; 69% | 70% | 60%; 57% | 63% |
| Coates, L. C. 2022 | 67% | 67% | 36% | 38% | - | - | 56% | 53% |
| Kristensen, L. E. 2022 | 61.5% | 60% | 30% | 61% | 99% | 99% | 65% | 65% |
| Ostor, A. 2022 | 65.6% | 72% | 17.9% | 26% | 94.6% | 95.0% | 49% | 45% |
| Philip J Mease 2021 | - | - | - | - | - | - | 56%; 60%; 64%; 54% | 59% |
