# Supplementary figures and images for: Efficacy and safety of IL-23 inhibitors in the treatment of psoriatic arthritis: a meta-analysis based on randomized controlled trials
Source: Immunol Res. 2023 Feb 22;71(4):505–15. doi: 10.1007/s12026-023-09366-4 (PMC10425519; doi:10.1007/s12026-023-09366-4)

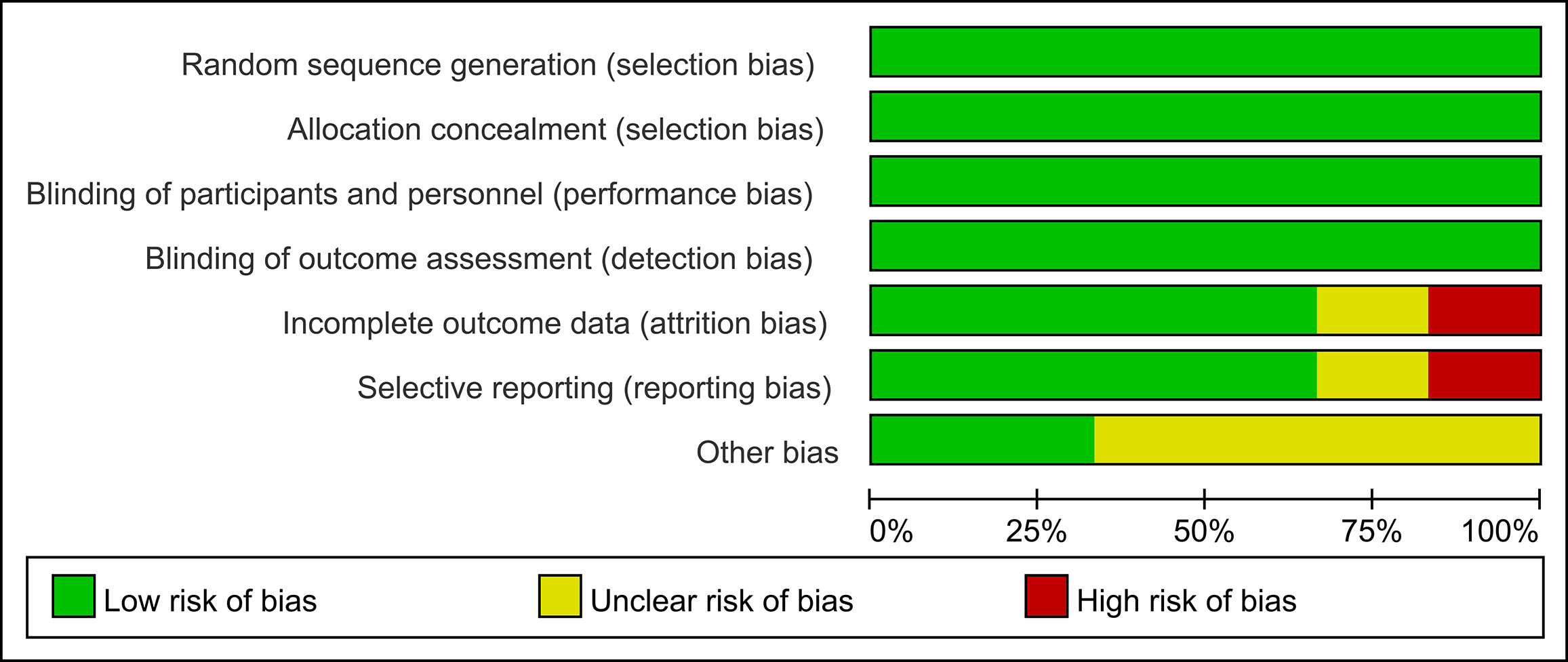

Supplement: Supplementary file 2 — Quality assessment of each study included in this meta-analysis [file 12026_2023_9366_Fig8_ESM.png]

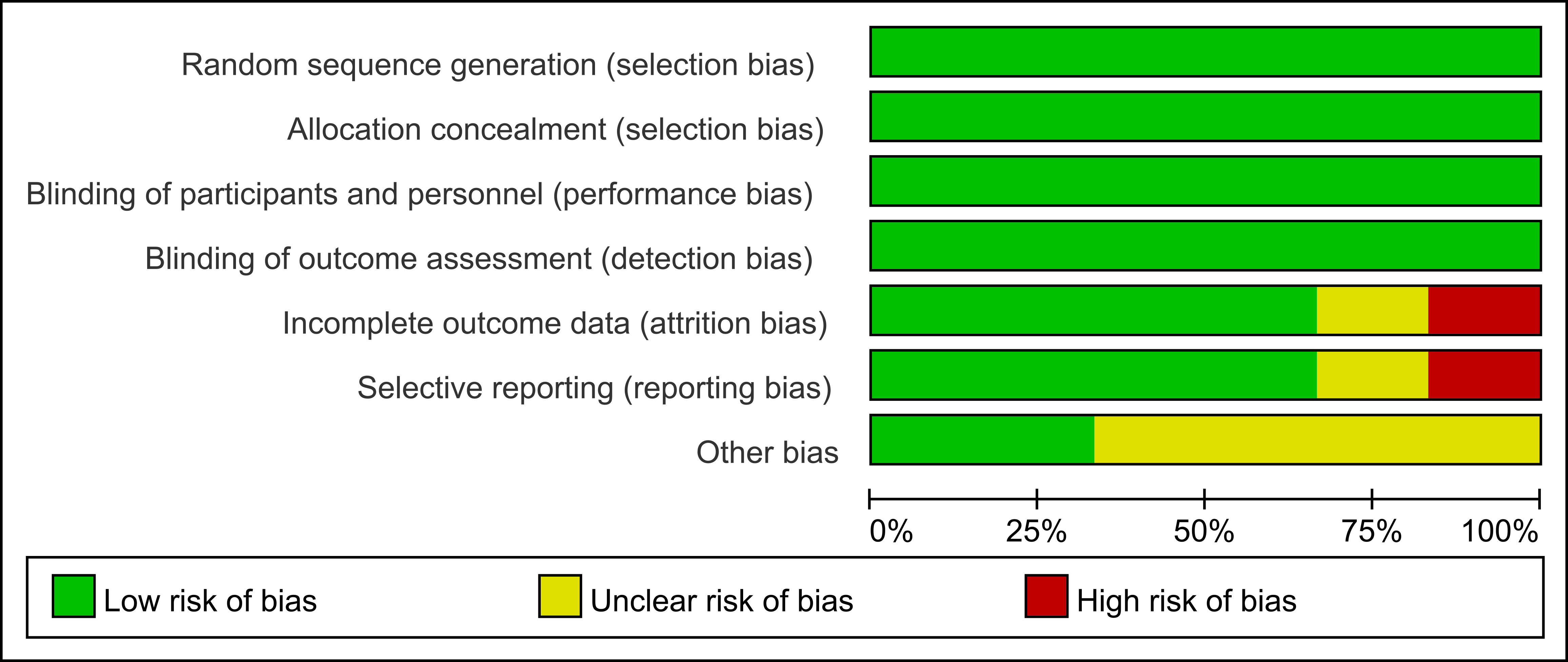

Supplement: Supplementary file 3 — High resolution image (TIF 440 kb) [file 12026_2023_9366_MOESM2_ESM.tif]

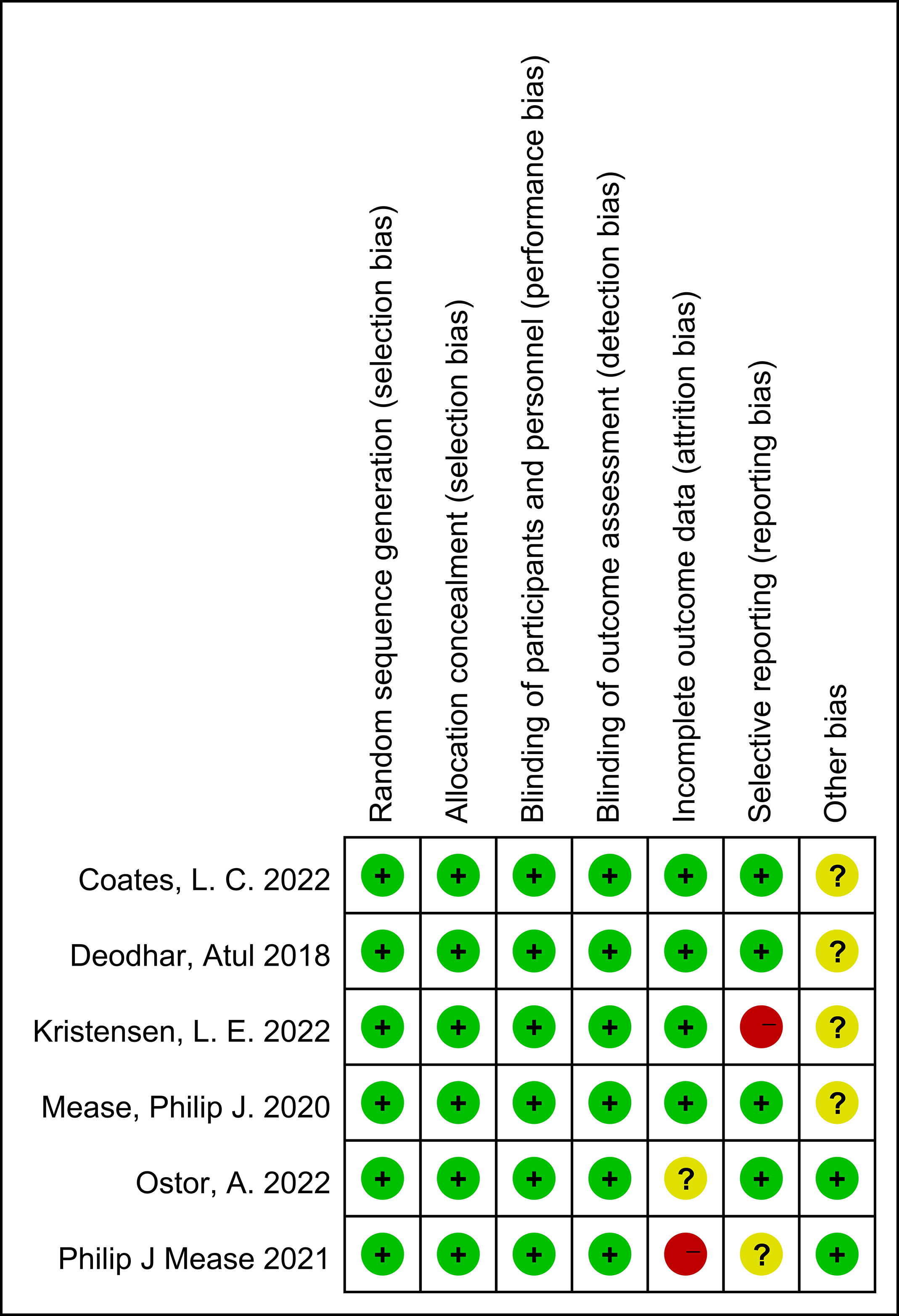

Supplement: Supplementary file 4 — Quality assessment of studies included in this meta-analysis (Risk of bias summary) [file 12026_2023_9366_Fig9_ESM.png]

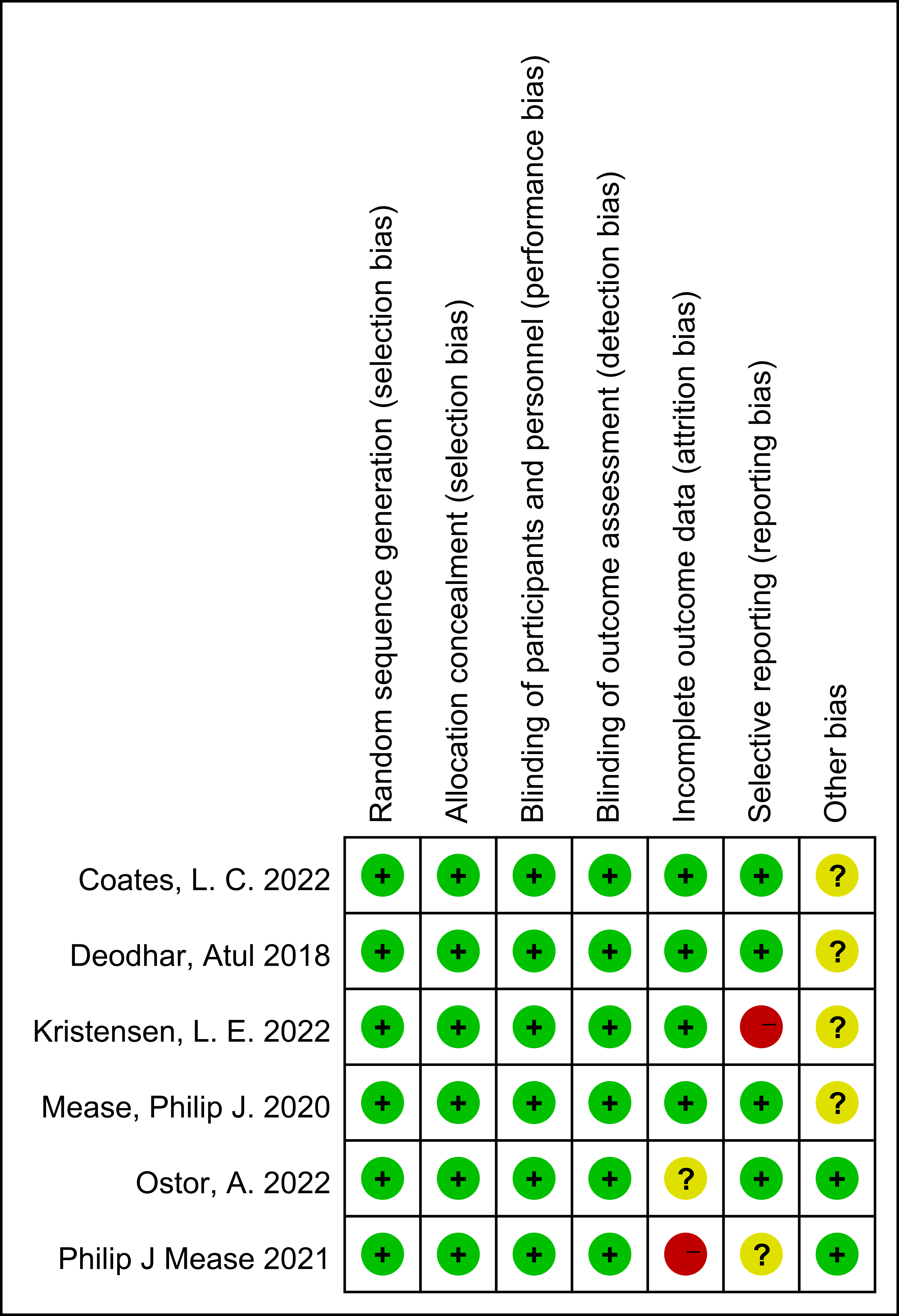

Supplement: Supplementary file 5 — High resolution image (TIF 1105 kb) [file 12026_2023_9366_MOESM3_ESM.tif]
